# 2-(4-Chloro­anilino)pyridine

**DOI:** 10.1107/S1600536808026317

**Published:** 2008-08-20

**Authors:** M. Zainal Abidin Fairuz, Zaharah Aiyub, Zanariah Abdullah, Seik Weng Ng

**Affiliations:** a Department of Chemistry, University of Malaya, 50603 Kuala Lumpur, Malaysia

## Abstract

The two aromatic rings of each of the four independent molecules in the asymmetric unit of the title compound, C_11_H_9_ClN_2_, are approximately coplanar; the four mol­ecules are arranged into two amino–pyridyl N—H⋯N hydrogen-bonded pairs. The structure has a 15% twin component related by a twofold rotation about [100].

## Related literature

The title compound exhibits fluorescence; see: Abdullah (2005 ▶); Kawai *et al.* (2001 ▶); Mohd Salleh *et al.* (2007 ▶). For the use of *PLATON* in the preparation of the diffraction data, see: Spek (2003 ▶).
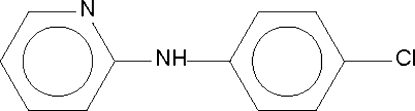


## Experimental

### 

#### Crystal data

C_11_H_9_ClN_2_
*M* *_r_* = 204.65
Triclinic, 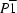

*a* = 7.3926 (3) Å
*b* = 15.3577 (5) Å
*c* = 17.6093 (6) Å
α = 73.723 (2)°
β = 87.360 (3)°
γ = 87.128 (3)°
*V* = 1915.6 (1) Å^3^
*Z* = 8
Mo *K*α radiation
μ = 0.35 mm^−1^
*T* = 100 (2) K
0.26 × 0.16 × 0.03 mm

#### Data collection

Bruker SMART APEX diffractometer
Absorption correction: multi-scan (*SADABS*; Sheldrick, 1996 ▶) *T*_min_ = 0.913, *T*_max_ = 0.989
14371 measured reflections
6679 independent reflections
4259 reflections with *I* > 2σ(*I*)
*R*_int_ = 0.068

#### Refinement

*R*[*F* ^2^ > 2σ(*F* ^2^)] = 0.073
*wR*(*F* ^2^) = 0.201
*S* = 1.04
6679 reflections
506 parameters
H-atom parameters constrained
Δρ_max_ = 0.52 e Å^−3^
Δρ_min_ = −0.52 e Å^−3^

### 

Data collection: *APEX2* (Bruker, 2007 ▶); cell refinement: *SAINT* (Bruker, 2007 ▶); data reduction: *SAINT*; program(s) used to solve structure: *SHELXS97* (Sheldrick, 2008 ▶); program(s) used to refine structure: *SHELXL97* (Sheldrick, 2008 ▶); molecular graphics: *X-SEED* (Barbour, 2001 ▶); software used to prepare material for publication: *publCIF* (Westrip, 2008 ▶).

## Supplementary Material

Crystal structure: contains datablocks global, I. DOI: 10.1107/S1600536808026317/pk2115sup1.cif

Structure factors: contains datablocks I. DOI: 10.1107/S1600536808026317/pk2115Isup2.hkl

Additional supplementary materials:  crystallographic information; 3D view; checkCIF report

## Figures and Tables

**Table 1:** Hydrogen-bond geometry (Å, °)

*D*—H⋯*A*	*D*—H	H⋯*A*	*D*⋯*A*	*D*—H⋯*A*
N2—H2*n*⋯N3	0.88	2.19	3.019 (5)	156
N4—H4*n*⋯N1	0.88	2.17	3.010 (5)	160
N6—H6*n*⋯N7	0.88	2.13	2.968 (5)	158
N8—H8*n*⋯N5	0.88	2.25	3.096 (5)	161

**Fig. 1.**
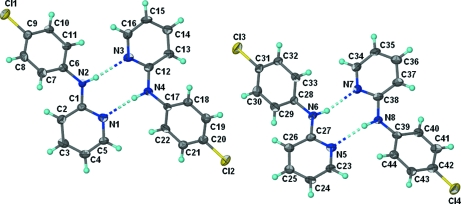
Thermal ellipsoid plot (Barbour, 2001) of the two pairs of hydrogen-bonded C11H9N2Cl molecules.

## supplementary crystallographic information

### Comment

The class of compounds represented by the title compound (Scheme I, Fig. 1) exhibit fluorescence (Abdullah, 2005; Kawai *et al.*, 2001; Mohd Salleh *et al.*, 2007). The compound crystallizes with four indepedent molecules; in each molecule, the two aromatic rings are approximately coplanar. The four molecules are arranged into two *H*–H_amino_–N_pyridyl_ hydrogen-bonded pairs.

### Experimental

2-Chloropyridine (0.5 ml, 5.28 mmol) and 4-chloroaniline (0.67 g, 5.28 mmol) were heated for 5 h. The mixture was cooled and extracted with ether (3 *x* 100 ml). The ether extract was washed with water and then dried over sodium sulfate. Evaporation of the solvent gave a purple colored powder. Recrystallization from chloroform yielded colorless prisms.

### Refinement

Carbon-bound H-atoms were placed in calculated positions (C—H 0.95 Å) and were included in the refinement using the riding model approximation, with *U*(H) fixed at 1.2*U*(C). The amino H-atoms were similarly treated as riding (N–H 0.88 Å).

The structure initially refined to a rather high *R* index of 8.26%, and the difference Fourier map showed relatively large peaks for an all-light atom structure, although none were larger than 1 *e* Å^-3^.  A preliminary check with the *TwinRotMat* routine of *PLATON* (Spek, 2003) showed strong evidence twofold twinning about [1 0 0].  Refinement against the *TwinRotMat*-generated data gave a lower *R* index of 7.26% along with a considerably flatter final difference Fourier map (no peak larger than *ca* 0.5 *e* Å^-3^). According to *TwinRotMat*, twinning should cause split reflections on the (*n*,*k*,*l*) layers with *n* = +/-1,2,3,4 although on some of these (*e.g.*, *n* =+/- 1,4) the spot splitting was marginal.  With *n* = -5,0,5 and on all (*h*,*n*,*l*) and (*h*,*k*,*n*) layers, the overlap was essentially perfect.

On the other hand, the reciprocal lattice diffraction data, when examined with the proprietary *RLATT* (Bruker, 2007) did not show any evidence of split reflections, most likely because the twin component is small.

### Crystal data

**Table:**

C_11_H_9_ClN_2_	*Z* = 8
*M**_r_* = 204.65	*F*_000_ = 848
Triclinic, *P*1	*D*_x_ = 1.419 Mg m^−^^3^
Hall symbol: -P 1	Mo *K*α radiation λ = 0.71073 Å
*a* = 7.3926 (3) Å	Cell parameters from 1607 reflections
*b* = 15.3577 (5) Å	θ = 2.7–22.1º
*c* = 17.6093 (6) Å	µ = 0.36 mm^−^^1^
α = 73.723 (2)º	*T* = 100 (2) K
β = 87.360 (3)º	Plate, colorless
γ = 87.128 (3)º	0.26 × 0.16 × 0.03 mm
*V* = 1915.6 (1) Å^3^	

### Data collection

**Table:**

Bruker SMART APEX diffractometer	6679 independent reflections
Radiation source: fine-focus sealed tube	4259 reflections with *I* > 2σ(*I*)
Monochromator: graphite	*R*_int_ = 0.068
*T* = 100(2) K	θ_max_ = 25.0º
ω scans	θ_min_ = 1.2º
Absorption correction: multi-scan(SADABS; Sheldrick, 1996)	*h* = −8→8
*T*_min_ = 0.913, *T*_max_ = 0.989	*k* = −18→18
14371 measured reflections	*l* = −20→20

### Refinement

**Table:**

Refinement on *F*^2^	Secondary atom site location: difference Fourier map
Least-squares matrix: full	Hydrogen site location: inferred from neighbouring sites
*R*[*F*^2^ > 2σ(*F*^2^)] = 0.073	H-atom parameters constrained
*wR*(*F*^2^) = 0.201	*w* = 1/[σ^2^(*F*_o_^2^) + (0.0956*P*)^2^] where *P* = (*F*_o_^2^ + 2*F*_c_^2^)/3
*S* = 1.04	(Δ/σ)_max_ = 0.001
6679 reflections	Δρ_max_ = 0.52 e Å^−^^3^
506 parameters	Δρ_min_ = −0.52 e Å^−^^3^
Primary atom site location: structure-invariant direct methods	Extinction correction: none

### Special details

**Table:**

Geometry. All e.s.d.'s (except the e.s.d. in the dihedral angle between two l.s. planes) are estimated using the full covariance matrix. The cell e.s.d.'s are taken into account individually in the estimation of e.s.d.'s in distances, angles and torsion angles; correlations between e.s.d.'s in cell parameters are only used when they are defined by crystal symmetry. An approximate (isotropic) treatment of cell e.s.d.'s is used for estimating e.s.d.'s involving l.s. planes.

### Fractional atomic coordinates and isotropic or equivalent isotropic displacement parameters (Å^2^)

**Table:**

	*x*	*y*	*z*	*U*_iso_*/*U*_eq_	
Cl1	0.63490 (18)	0.33288 (8)	0.13449 (6)	0.0313 (3)	
Cl2	0.86940 (19)	0.47050 (8)	0.93970 (7)	0.0348 (4)	
Cl3	0.85771 (18)	0.82618 (8)	0.64005 (7)	0.0323 (3)	
Cl4	0.68780 (19)	0.97391 (8)	1.44898 (7)	0.0344 (4)	
N1	0.7638 (5)	0.2816 (2)	0.6012 (2)	0.0235 (9)	
N2	0.7050 (5)	0.3289 (2)	0.4689 (2)	0.0241 (9)	
H2N	0.6767	0.3812	0.4785	0.029*	
N3	0.7168 (6)	0.5220 (2)	0.4778 (2)	0.0274 (10)	
N4	0.8145 (6)	0.4735 (2)	0.6050 (2)	0.0266 (10)	
H4N	0.8259	0.4192	0.5975	0.032*	
N5	0.6763 (5)	0.7791 (2)	1.1073 (2)	0.0242 (9)	
N6	0.7641 (5)	0.8237 (2)	0.97657 (19)	0.0232 (9)	
H6N	0.7937	0.8742	0.9864	0.028*	
N7	0.7819 (6)	1.0150 (2)	0.9807 (2)	0.0264 (10)	
N8	0.6946 (6)	0.9785 (2)	1.1120 (2)	0.0253 (10)	
H8N	0.6648	0.9263	1.1056	0.030*	
C1	0.7593 (6)	0.2583 (3)	0.5324 (2)	0.0198 (10)	
C2	0.8080 (6)	0.1703 (3)	0.5277 (3)	0.0231 (11)	
H2	0.8022	0.1552	0.4792	0.028*	
C3	0.8645 (7)	0.1062 (3)	0.5954 (3)	0.0268 (11)	
H3	0.8996	0.0465	0.5934	0.032*	
C4	0.8702 (7)	0.1288 (3)	0.6660 (3)	0.0256 (11)	
H4	0.9076	0.0854	0.7131	0.031*	
C5	0.8195 (7)	0.2166 (3)	0.6653 (3)	0.0241 (11)	
H5	0.8244	0.2324	0.7136	0.029*	
C6	0.6904 (7)	0.3257 (3)	0.3904 (2)	0.0234 (11)	
C7	0.6014 (6)	0.2570 (3)	0.3717 (3)	0.0224 (11)	
H7	0.5506	0.2092	0.4126	0.027*	
C8	0.5873 (6)	0.2588 (3)	0.2926 (3)	0.0243 (11)	
H8	0.5312	0.2108	0.2795	0.029*	
C9	0.6547 (6)	0.3301 (3)	0.2336 (2)	0.0221 (11)	
C10	0.7396 (6)	0.4008 (3)	0.2509 (2)	0.0219 (11)	
H10	0.7847	0.4500	0.2098	0.026*	
C11	0.7564 (7)	0.3970 (3)	0.3299 (2)	0.0236 (11)	
H11	0.8145	0.4445	0.3428	0.028*	
C12	0.7827 (6)	0.5452 (3)	0.5387 (2)	0.0225 (11)	
C13	0.8204 (6)	0.6354 (3)	0.5341 (3)	0.0242 (11)	
H13	0.8699	0.6502	0.5775	0.029*	
C14	0.7839 (6)	0.7019 (3)	0.4651 (2)	0.0250 (11)	
H14	0.8077	0.7635	0.4604	0.030*	
C15	0.7119 (7)	0.6780 (3)	0.4026 (3)	0.0284 (12)	
H15	0.6839	0.7228	0.3547	0.034*	
C16	0.6823 (7)	0.5882 (3)	0.4117 (3)	0.0267 (11)	
H16	0.6345	0.5720	0.3685	0.032*	
C17	0.8306 (6)	0.4777 (3)	0.6828 (2)	0.0197 (10)	
C18	0.7407 (6)	0.5441 (3)	0.7125 (2)	0.0225 (11)	
H18	0.6689	0.5906	0.6785	0.027*	
C19	0.7548 (6)	0.5429 (3)	0.7910 (2)	0.0234 (11)	
H19	0.6954	0.5889	0.8105	0.028*	
C20	0.8569 (6)	0.4734 (3)	0.8409 (2)	0.0232 (11)	
C21	0.9467 (6)	0.4064 (3)	0.8133 (2)	0.0235 (11)	
H21	1.0167	0.3594	0.8477	0.028*	
C22	0.9327 (7)	0.4092 (3)	0.7348 (3)	0.0242 (11)	
H22	0.9938	0.3634	0.7155	0.029*	
C23	0.6090 (7)	0.7168 (3)	1.1698 (3)	0.0258 (11)	
H23	0.5938	0.7324	1.2183	0.031*	
C24	0.5600 (7)	0.6317 (3)	1.1694 (3)	0.0270 (12)	
H24	0.5098	0.5904	1.2154	0.032*	
C25	0.5872 (7)	0.6088 (3)	1.0989 (2)	0.0259 (12)	
H25	0.5581	0.5502	1.0963	0.031*	
C26	0.6557 (7)	0.6702 (3)	1.0330 (3)	0.0257 (11)	
H26	0.6731	0.6554	0.9843	0.031*	
C27	0.7002 (6)	0.7565 (3)	1.0396 (2)	0.0230 (11)	
C28	0.7872 (6)	0.8202 (3)	0.8975 (2)	0.0179 (10)	
C29	0.8870 (6)	0.7517 (3)	0.8758 (3)	0.0235 (11)	
H29	0.9420	0.7036	0.9153	0.028*	
C30	0.9073 (6)	0.7526 (3)	0.7969 (3)	0.0252 (11)	
H30	0.9730	0.7047	0.7823	0.030*	
C31	0.8304 (6)	0.8243 (3)	0.7398 (2)	0.0221 (11)	
C32	0.7343 (6)	0.8940 (3)	0.7592 (2)	0.0212 (11)	
H32	0.6846	0.9432	0.7190	0.025*	
C33	0.7101 (6)	0.8919 (3)	0.8391 (2)	0.0203 (10)	
H33	0.6414	0.9391	0.8535	0.024*	
C34	0.8266 (7)	1.0766 (3)	0.9131 (3)	0.0280 (12)	
H34	0.8587	1.0553	0.8684	0.034*	
C35	0.8294 (7)	1.1685 (3)	0.9036 (3)	0.0276 (12)	
H35	0.8688	1.2092	0.8550	0.033*	
C36	0.7723 (7)	1.1994 (3)	0.9681 (3)	0.0307 (12)	
H36	0.7666	1.2626	0.9637	0.037*	
C37	0.7243 (6)	1.1380 (3)	1.0383 (3)	0.0251 (11)	
H37	0.6846	1.1580	1.0829	0.030*	
C38	0.7346 (7)	1.0458 (3)	1.0431 (2)	0.0239 (11)	
C39	0.6953 (6)	0.9824 (3)	1.1897 (2)	0.0213 (10)	
C40	0.7925 (7)	1.0447 (3)	1.2151 (2)	0.0240 (11)	
H40	0.8622	1.0883	1.1778	0.029*	
C41	0.7873 (6)	1.0427 (3)	1.2940 (3)	0.0248 (11)	
H41	0.8508	1.0860	1.3107	0.030*	
C42	0.6890 (6)	0.9773 (3)	1.3491 (3)	0.0239 (11)	
C43	0.5945 (7)	0.9148 (3)	1.3258 (2)	0.0234 (11)	
H43	0.5293	0.8697	1.3637	0.028*	
C44	0.5958 (6)	0.9185 (3)	1.2462 (2)	0.0230 (11)	
H44	0.5275	0.8767	1.2298	0.028*	

### Atomic displacement parameters (Å^2^)

**Table:**

	*U*^11^	*U*^22^	*U*^33^	*U*^12^	*U*^13^	*U*^23^
Cl1	0.0369 (8)	0.0382 (7)	0.0208 (6)	0.0045 (6)	−0.0054 (5)	−0.0120 (5)
Cl2	0.0479 (9)	0.0381 (7)	0.0209 (6)	0.0003 (6)	−0.0111 (6)	−0.0113 (5)
Cl3	0.0375 (8)	0.0411 (7)	0.0217 (6)	−0.0116 (6)	0.0039 (5)	−0.0132 (5)
Cl4	0.0421 (9)	0.0412 (7)	0.0214 (6)	−0.0039 (6)	0.0045 (6)	−0.0114 (5)
N1	0.026 (2)	0.0204 (19)	0.022 (2)	−0.0022 (17)	−0.0010 (17)	−0.0023 (16)
N2	0.040 (3)	0.0166 (18)	0.0154 (19)	−0.0009 (18)	−0.0052 (17)	−0.0041 (15)
N3	0.038 (3)	0.022 (2)	0.020 (2)	0.0031 (19)	−0.0047 (18)	−0.0027 (16)
N4	0.043 (3)	0.0176 (19)	0.0169 (19)	0.0005 (19)	−0.0004 (18)	−0.0021 (15)
N5	0.028 (2)	0.0214 (19)	0.021 (2)	−0.0015 (18)	−0.0023 (17)	−0.0025 (16)
N6	0.034 (3)	0.0211 (19)	0.0137 (18)	−0.0044 (18)	0.0002 (17)	−0.0037 (15)
N7	0.039 (3)	0.023 (2)	0.017 (2)	−0.0059 (19)	−0.0035 (18)	−0.0050 (16)
N8	0.042 (3)	0.0178 (19)	0.0151 (19)	−0.0064 (18)	−0.0036 (17)	−0.0014 (15)
C1	0.021 (3)	0.021 (2)	0.017 (2)	−0.008 (2)	−0.0024 (19)	−0.0021 (18)
C2	0.028 (3)	0.025 (2)	0.017 (2)	−0.004 (2)	0.000 (2)	−0.0063 (19)
C3	0.027 (3)	0.021 (2)	0.030 (3)	0.000 (2)	−0.001 (2)	−0.002 (2)
C4	0.030 (3)	0.023 (2)	0.021 (2)	−0.004 (2)	−0.005 (2)	0.0004 (19)
C5	0.032 (3)	0.021 (2)	0.017 (2)	−0.004 (2)	−0.006 (2)	0.0001 (18)
C6	0.031 (3)	0.020 (2)	0.019 (2)	0.000 (2)	−0.007 (2)	−0.0049 (19)
C7	0.020 (3)	0.022 (2)	0.025 (2)	−0.002 (2)	−0.002 (2)	−0.0064 (19)
C8	0.024 (3)	0.020 (2)	0.032 (3)	0.004 (2)	−0.007 (2)	−0.012 (2)
C9	0.024 (3)	0.023 (2)	0.020 (2)	0.003 (2)	−0.005 (2)	−0.0074 (18)
C10	0.025 (3)	0.019 (2)	0.020 (2)	0.001 (2)	−0.002 (2)	−0.0016 (18)
C11	0.032 (3)	0.019 (2)	0.019 (2)	−0.003 (2)	−0.003 (2)	−0.0032 (18)
C12	0.026 (3)	0.025 (2)	0.016 (2)	−0.004 (2)	0.003 (2)	−0.0036 (18)
C13	0.028 (3)	0.022 (2)	0.021 (2)	0.000 (2)	0.000 (2)	−0.0029 (19)
C14	0.028 (3)	0.022 (2)	0.023 (2)	−0.004 (2)	−0.001 (2)	−0.0031 (19)
C15	0.038 (3)	0.024 (2)	0.018 (2)	0.007 (2)	−0.002 (2)	0.0020 (19)
C16	0.032 (3)	0.027 (3)	0.022 (2)	0.003 (2)	−0.007 (2)	−0.008 (2)
C17	0.027 (3)	0.014 (2)	0.017 (2)	−0.003 (2)	0.002 (2)	−0.0027 (17)
C18	0.026 (3)	0.020 (2)	0.021 (2)	−0.003 (2)	−0.003 (2)	−0.0026 (18)
C19	0.023 (3)	0.022 (2)	0.025 (2)	−0.005 (2)	0.001 (2)	−0.0060 (19)
C20	0.024 (3)	0.025 (2)	0.019 (2)	−0.011 (2)	−0.001 (2)	−0.0030 (19)
C21	0.027 (3)	0.022 (2)	0.020 (2)	−0.003 (2)	−0.005 (2)	−0.0028 (19)
C22	0.032 (3)	0.013 (2)	0.025 (2)	−0.001 (2)	0.000 (2)	−0.0026 (18)
C23	0.037 (3)	0.020 (2)	0.018 (2)	0.000 (2)	0.000 (2)	−0.0022 (19)
C24	0.034 (3)	0.021 (2)	0.021 (2)	−0.003 (2)	0.001 (2)	0.0028 (19)
C25	0.031 (3)	0.021 (2)	0.025 (3)	−0.006 (2)	−0.008 (2)	−0.0032 (19)
C26	0.033 (3)	0.020 (2)	0.022 (2)	0.001 (2)	−0.006 (2)	−0.0027 (19)
C27	0.026 (3)	0.023 (2)	0.018 (2)	−0.001 (2)	−0.007 (2)	−0.0010 (19)
C28	0.020 (3)	0.019 (2)	0.016 (2)	−0.0062 (19)	0.0003 (19)	−0.0056 (17)
C29	0.024 (3)	0.019 (2)	0.027 (2)	−0.001 (2)	−0.003 (2)	−0.0050 (19)
C30	0.024 (3)	0.027 (2)	0.027 (3)	−0.003 (2)	0.004 (2)	−0.011 (2)
C31	0.027 (3)	0.025 (2)	0.015 (2)	−0.008 (2)	0.004 (2)	−0.0062 (18)
C32	0.025 (3)	0.021 (2)	0.017 (2)	−0.005 (2)	−0.0010 (19)	−0.0025 (18)
C33	0.020 (3)	0.017 (2)	0.023 (2)	−0.0011 (19)	0.000 (2)	−0.0051 (18)
C34	0.040 (3)	0.024 (2)	0.019 (2)	−0.005 (2)	−0.004 (2)	−0.0048 (19)
C35	0.038 (3)	0.025 (2)	0.015 (2)	−0.004 (2)	−0.005 (2)	0.0011 (19)
C36	0.039 (3)	0.023 (2)	0.028 (3)	−0.003 (2)	−0.010 (2)	−0.001 (2)
C37	0.027 (3)	0.026 (2)	0.020 (2)	−0.001 (2)	−0.005 (2)	−0.0006 (19)
C38	0.029 (3)	0.023 (2)	0.017 (2)	−0.003 (2)	−0.004 (2)	−0.0005 (19)
C39	0.025 (3)	0.018 (2)	0.018 (2)	0.004 (2)	−0.003 (2)	−0.0002 (18)
C40	0.028 (3)	0.020 (2)	0.022 (2)	−0.003 (2)	−0.003 (2)	−0.0026 (19)
C41	0.021 (3)	0.025 (2)	0.025 (3)	−0.002 (2)	0.000 (2)	−0.002 (2)
C42	0.023 (3)	0.026 (2)	0.020 (2)	0.006 (2)	0.001 (2)	−0.0024 (19)
C43	0.028 (3)	0.020 (2)	0.020 (2)	−0.004 (2)	0.005 (2)	−0.0034 (19)
C44	0.027 (3)	0.019 (2)	0.022 (2)	−0.005 (2)	−0.004 (2)	−0.0028 (18)

### Geometric parameters (Å, °)

**Table:**

Cl1—C9	1.747 (4)	C15—H15	0.9500
Cl2—C20	1.734 (4)	C16—H16	0.9500
Cl3—C31	1.751 (4)	C17—C18	1.397 (6)
Cl4—C42	1.744 (5)	C17—C22	1.400 (6)
N1—C5	1.345 (5)	C18—C19	1.385 (6)
N1—C1	1.359 (5)	C18—H18	0.9500
N2—C1	1.380 (5)	C19—C20	1.391 (6)
N2—C6	1.406 (5)	C19—H19	0.9500
N2—H2N	0.8800	C20—C21	1.386 (7)
N3—C16	1.338 (5)	C21—C22	1.378 (6)
N3—C12	1.340 (6)	C21—H21	0.9500
N4—C12	1.381 (5)	C22—H22	0.9500
N4—C17	1.401 (5)	C23—C24	1.376 (6)
N4—H4N	0.8800	C23—H23	0.9500
N5—C27	1.334 (6)	C24—C25	1.385 (6)
N5—C23	1.335 (5)	C24—H24	0.9500
N6—C27	1.370 (5)	C25—C26	1.368 (6)
N6—C28	1.411 (5)	C25—H25	0.9500
N6—H6N	0.8800	C26—C27	1.419 (6)
N7—C34	1.336 (5)	C26—H26	0.9500
N7—C38	1.340 (6)	C28—C29	1.385 (6)
N8—C39	1.386 (5)	C28—C33	1.397 (6)
N8—C38	1.387 (5)	C29—C30	1.387 (6)
N8—H8N	0.8800	C29—H29	0.9500
C1—C2	1.405 (6)	C30—C31	1.385 (6)
C2—C3	1.382 (6)	C30—H30	0.9500
C2—H2	0.9500	C31—C32	1.369 (6)
C3—C4	1.384 (6)	C32—C33	1.401 (6)
C3—H3	0.9500	C32—H32	0.9500
C4—C5	1.378 (6)	C33—H33	0.9500
C4—H4	0.9500	C34—C35	1.376 (6)
C5—H5	0.9500	C34—H34	0.9500
C6—C11	1.387 (5)	C35—C36	1.389 (7)
C6—C7	1.393 (6)	C35—H35	0.9500
C7—C8	1.394 (6)	C36—C37	1.373 (6)
C7—H7	0.9500	C36—H36	0.9500
C8—C9	1.377 (6)	C37—C38	1.392 (6)
C8—H8	0.9500	C37—H37	0.9500
C9—C10	1.391 (6)	C39—C44	1.397 (6)
C10—C11	1.387 (6)	C39—C40	1.403 (6)
C10—H10	0.9500	C40—C41	1.381 (6)
C11—H11	0.9500	C40—H40	0.9500
C12—C13	1.406 (6)	C41—C42	1.393 (6)
C13—C14	1.377 (6)	C41—H41	0.9500
C13—H13	0.9500	C42—C43	1.376 (6)
C14—C15	1.390 (6)	C43—C44	1.386 (6)
C14—H14	0.9500	C43—H43	0.9500
C15—C16	1.371 (6)	C44—H44	0.9500
			
C5—N1—C1	117.0 (4)	C21—C20—Cl2	119.7 (3)
C1—N2—C6	126.6 (4)	C19—C20—Cl2	119.1 (4)
C1—N2—H2N	116.7	C22—C21—C20	118.7 (4)
C6—N2—H2N	116.7	C22—C21—H21	120.6
C16—N3—C12	117.9 (4)	C20—C21—H21	120.6
C12—N4—C17	127.2 (4)	C21—C22—C17	121.8 (5)
C12—N4—H4N	116.4	C21—C22—H22	119.1
C17—N4—H4N	116.4	C17—C22—H22	119.1
C27—N5—C23	117.4 (4)	N5—C23—C24	125.0 (4)
C27—N6—C28	126.6 (4)	N5—C23—H23	117.5
C27—N6—H6N	116.7	C24—C23—H23	117.5
C28—N6—H6N	116.7	C23—C24—C25	117.0 (4)
C34—N7—C38	117.1 (4)	C23—C24—H24	121.5
C39—N8—C38	128.7 (4)	C25—C24—H24	121.5
C39—N8—H8N	115.7	C26—C25—C24	120.4 (4)
C38—N8—H8N	115.7	C26—C25—H25	119.8
N1—C1—N2	113.7 (4)	C24—C25—H25	119.8
N1—C1—C2	122.1 (4)	C25—C26—C27	118.2 (4)
N2—C1—C2	124.2 (4)	C25—C26—H26	120.9
C3—C2—C1	118.5 (4)	C27—C26—H26	120.9
C3—C2—H2	120.7	N5—C27—N6	115.1 (4)
C1—C2—H2	120.7	N5—C27—C26	122.1 (4)
C2—C3—C4	120.2 (4)	N6—C27—C26	122.7 (4)
C2—C3—H3	119.9	C29—C28—C33	119.6 (4)
C4—C3—H3	119.9	C29—C28—N6	123.2 (4)
C5—C4—C3	117.5 (4)	C33—C28—N6	117.2 (4)
C5—C4—H4	121.2	C28—C29—C30	120.6 (4)
C3—C4—H4	121.2	C28—C29—H29	119.7
N1—C5—C4	124.7 (4)	C30—C29—H29	119.7
N1—C5—H5	117.6	C31—C30—C29	119.0 (5)
C4—C5—H5	117.6	C31—C30—H30	120.5
C11—C6—C7	119.4 (4)	C29—C30—H30	120.5
C11—C6—N2	118.3 (4)	C32—C31—C30	121.8 (4)
C7—C6—N2	122.1 (4)	C32—C31—Cl3	119.1 (3)
C6—C7—C8	119.5 (4)	C30—C31—Cl3	119.1 (4)
C6—C7—H7	120.3	C31—C32—C33	119.2 (4)
C8—C7—H7	120.3	C31—C32—H32	120.4
C9—C8—C7	120.0 (4)	C33—C32—H32	120.4
C9—C8—H8	120.0	C28—C33—C32	119.9 (4)
C7—C8—H8	120.0	C28—C33—H33	120.1
C8—C9—C10	121.5 (4)	C32—C33—H33	120.1
C8—C9—Cl1	119.9 (3)	N7—C34—C35	124.7 (5)
C10—C9—Cl1	118.6 (3)	N7—C34—H34	117.6
C11—C10—C9	117.9 (4)	C35—C34—H34	117.6
C11—C10—H10	121.0	C34—C35—C36	117.2 (4)
C9—C10—H10	121.0	C34—C35—H35	121.4
C10—C11—C6	121.7 (4)	C36—C35—H35	121.4
C10—C11—H11	119.2	C37—C36—C35	119.5 (4)
C6—C11—H11	119.2	C37—C36—H36	120.2
N3—C12—N4	114.8 (4)	C35—C36—H36	120.2
N3—C12—C13	122.1 (4)	C36—C37—C38	119.0 (5)
N4—C12—C13	123.1 (4)	C36—C37—H37	120.5
C14—C13—C12	118.5 (4)	C38—C37—H37	120.5
C14—C13—H13	120.7	N7—C38—N8	114.3 (4)
C12—C13—H13	120.7	N7—C38—C37	122.4 (4)
C13—C14—C15	119.3 (4)	N8—C38—C37	123.2 (4)
C13—C14—H14	120.4	N8—C39—C44	117.4 (4)
C15—C14—H14	120.4	N8—C39—C40	124.2 (4)
C16—C15—C14	118.3 (4)	C44—C39—C40	118.3 (4)
C16—C15—H15	120.8	C41—C40—C39	120.2 (4)
C14—C15—H15	120.8	C41—C40—H40	119.9
N3—C16—C15	123.8 (4)	C39—C40—H40	119.9
N3—C16—H16	118.1	C40—C41—C42	120.0 (4)
C15—C16—H16	118.1	C40—C41—H41	120.0
C18—C17—C22	118.2 (4)	C42—C41—H41	120.0
C18—C17—N4	123.1 (4)	C43—C42—C41	120.8 (4)
C22—C17—N4	118.6 (4)	C43—C42—Cl4	119.6 (3)
C19—C18—C17	120.8 (4)	C41—C42—Cl4	119.6 (4)
C19—C18—H18	119.6	C42—C43—C44	119.0 (4)
C17—C18—H18	119.6	C42—C43—H43	120.5
C18—C19—C20	119.2 (5)	C44—C43—H43	120.5
C18—C19—H19	120.4	C43—C44—C39	121.6 (4)
C20—C19—H19	120.4	C43—C44—H44	119.2
C21—C20—C19	121.2 (4)	C39—C44—H44	119.2
			
C5—N1—C1—N2	−178.5 (4)	C27—N5—C23—C24	−1.1 (7)
C5—N1—C1—C2	0.8 (6)	N5—C23—C24—C25	1.6 (8)
C6—N2—C1—N1	178.8 (4)	C23—C24—C25—C26	−1.5 (7)
C6—N2—C1—C2	−0.5 (7)	C24—C25—C26—C27	1.0 (7)
N1—C1—C2—C3	−0.9 (7)	C23—N5—C27—N6	178.0 (4)
N2—C1—C2—C3	178.3 (4)	C23—N5—C27—C26	0.4 (7)
C1—C2—C3—C4	0.8 (7)	C28—N6—C27—N5	−174.5 (4)
C2—C3—C4—C5	−0.6 (7)	C28—N6—C27—C26	3.1 (7)
C1—N1—C5—C4	−0.6 (7)	C25—C26—C27—N5	−0.4 (7)
C3—C4—C5—N1	0.5 (7)	C25—C26—C27—N6	−177.8 (4)
C1—N2—C6—C11	−136.0 (5)	C27—N6—C28—C29	−54.0 (6)
C1—N2—C6—C7	48.5 (7)	C27—N6—C28—C33	128.5 (5)
C11—C6—C7—C8	2.9 (7)	C33—C28—C29—C30	−1.5 (6)
N2—C6—C7—C8	178.4 (4)	N6—C28—C29—C30	−179.0 (4)
C6—C7—C8—C9	−2.6 (7)	C28—C29—C30—C31	1.7 (6)
C7—C8—C9—C10	0.8 (7)	C29—C30—C31—C32	−0.3 (7)
C7—C8—C9—Cl1	−179.3 (4)	C29—C30—C31—Cl3	179.4 (3)
C8—C9—C10—C11	0.6 (7)	C30—C31—C32—C33	−1.2 (7)
Cl1—C9—C10—C11	−179.2 (4)	Cl3—C31—C32—C33	179.1 (3)
C9—C10—C11—C6	−0.3 (7)	C29—C28—C33—C32	0.0 (6)
C7—C6—C11—C10	−1.5 (7)	N6—C28—C33—C32	177.6 (4)
N2—C6—C11—C10	−177.1 (4)	C31—C32—C33—C28	1.4 (6)
C16—N3—C12—N4	179.8 (4)	C38—N7—C34—C35	0.9 (7)
C16—N3—C12—C13	−1.8 (7)	N7—C34—C35—C36	−3.6 (8)
C17—N4—C12—N3	−159.7 (5)	C34—C35—C36—C37	2.9 (7)
C17—N4—C12—C13	21.9 (8)	C35—C36—C37—C38	0.2 (7)
N3—C12—C13—C14	1.6 (8)	C34—N7—C38—N8	−178.3 (4)
N4—C12—C13—C14	179.9 (5)	C34—N7—C38—C37	2.6 (7)
C12—C13—C14—C15	−0.2 (7)	C39—N8—C38—N7	158.5 (5)
C13—C14—C15—C16	−1.0 (8)	C39—N8—C38—C37	−22.4 (8)
C12—N3—C16—C15	0.6 (8)	C36—C37—C38—N7	−3.1 (7)
C14—C15—C16—N3	0.8 (8)	C36—C37—C38—N8	177.9 (5)
C12—N4—C17—C18	30.0 (7)	C38—N8—C39—C44	159.5 (5)
C12—N4—C17—C22	−153.9 (4)	C38—N8—C39—C40	−22.0 (8)
C22—C17—C18—C19	1.1 (6)	N8—C39—C40—C41	−179.1 (5)
N4—C17—C18—C19	177.1 (4)	C44—C39—C40—C41	−0.6 (7)
C17—C18—C19—C20	−1.4 (6)	C39—C40—C41—C42	1.6 (7)
C18—C19—C20—C21	1.0 (6)	C40—C41—C42—C43	−0.8 (7)
C18—C19—C20—Cl2	−178.3 (3)	C40—C41—C42—Cl4	178.5 (4)
C19—C20—C21—C22	−0.3 (6)	C41—C42—C43—C44	−1.0 (7)
Cl2—C20—C21—C22	179.0 (3)	Cl4—C42—C43—C44	179.7 (4)
C20—C21—C22—C17	0.0 (7)	C42—C43—C44—C39	2.1 (7)
C18—C17—C22—C21	−0.4 (6)	N8—C39—C44—C43	177.3 (4)
N4—C17—C22—C21	−176.6 (4)	C40—C39—C44—C43	−1.2 (7)

### Hydrogen-bond geometry (Å, °)

**Table:**

*D*—H···*A*	*D*—H	H···*A*	*D*···*A*	*D*—H···*A*
N2—H2n···N3	0.88	2.19	3.019 (5)	156
N4—H4n···N1	0.88	2.17	3.010 (5)	160
N6—H6n···N7	0.88	2.13	2.968 (5)	158
N8—H8n···N5	0.88	2.25	3.096 (5)	161
